# Inequities in utilization of reproductive and maternal health services in Ethiopia

**DOI:** 10.1186/s12939-017-0602-2

**Published:** 2017-06-19

**Authors:** Firew Tekle Bobo, Elias Ali Yesuf, Mirkuzie Woldie

**Affiliations:** 1 Department of Public Health, College of Health sciences; Wollega University, Nekemte, Ethiopia; 2 Department of Health Economics, Management, and Policy, Faculty of Public Health; Jimma University, Jimma, Ethiopia; 3 CIH-LMU Center for International Health, Ludwig-Maximilians-Universität München, Munich, Germany

**Keywords:** Inequity, Modern contraceptive, Antenatal care, Postnatal care, Birth in the facility, Ethiopia

## Abstract

**Background:**

Disparities in health services utilization within and between regional states of countries with diverse socio-cultural and economic conditions such as Ethiopia is a frequent encounter. Understanding and taking measures to address unnecessary and avoidable differences in the use of reproductive and maternal health services is a key concern in Ethiopia. The aim of the study was to examine degree of equity in reproductive and maternal health services utilization in Ethiopia.

**Method:**

Data from Ethiopia demographic health survey 2014 was analyzed. We assessed inequities in utilization of modern contraceptive methods, antenatal care, facility based delivery and postnatal checkup. Four standard equity measurement methods were used; equity gaps, rate-ratios, concertation curve and concentration index.

**Results:**

Inequities in service utilization were exhibited favoring women in developed regions, urban residents, most educated and the wealthy. Antenatal care by skilled provider was three times higher among women with post-secondary education than mothers with no education. Women in the highest wealth quantile had about 12 times higher skilled birth attendance than those in lowest wealth quantile. The rate of postnatal care use among urban resident was about 6 times that of women in rural area. Use of modern contraceptive methods was more equitably utilized service while, birth at health facility was less equitable across all economic levels, favoring the wealthy.

**Conclusion:**

Considerable inequity between and within regions of Ethiopia in the use of maternal health services was demonstrated. Strategically targeting social determinants of health with special emphasis to women education and economic empowerment will substantially contribute for altering the current situation favorably.

## Background

Equity in the health sector has long been regarded as an important objective of the health system [1, 2]. The poor tend to suffer greater rates of morbidity and mortality than do the rich. Despite the fact having higher level need for health services, they often utilize less than the rich [2, 3].

In developing countries, maternal health service utilization has improved over a time. However, many studies demonstrate the presence of significant inequalities based on urban/rural, education, socioeconomic and regional differences [4–6].

Use of modern contraceptive methods, antenatal care (ANC), skilled birth attendance and postnatal care (PNC) are among the key maternal health interventions to have made undeniable differences in reducing maternal mortality worldwide [7, 8].

However, there is a limited use of those key interventions in developing countries, and are reported to have variations among population groups [8, 9]. Health status in Ethiopia has dramatic improvements in recent decades [10–12]. The gains have been as a result of improvements in utilization of health care services amongst disadvantaged groups, particularly those living in rural areas [2, 13]. Despite this progress, substantial inequalities still exist in health outcomes based on differences in economic status, education, place of residence and sex [14].

Disparities in reproductive and maternal health service utilization can be attributed to limitations in supply side associated to access of the service, quality of the service and affordability of the services [11, 15]. Accessibility of services in Ghana was largely observed favoring women residing in urban area than rural area, best performing region than the worst, highest wealth quantile than the lowest and at least secondary education than those with no formal education [16].

Limitations to the demand side can similarly affect utilization of maternal health services, and may operate at the individual, household and community levels. Factors at the individual level may include, age, income, education, culture and women’s autonomy which could all largely contribute to women’s capability to seek care [3, 5, 9, 17].

In Ethiopian context, both supply and demand sides significantly contribute to differences in maternal health service utilization. Major factors making things difficult for Ethiopian women to seek services may include indirect cost factors, cultural barriers, transport, perceived quality of care and autonomy demanding urgent interventions [15, 18].

Accelerated Expansion of Primary Health Care Coverage through health posts, is one of the initiatives undertaken by Ethiopian federal ministry of health to deliver preventive maternal and child health interventions mainly to the rural and impoverished parts of the population [19]. The country recently devised a new 5-year plan for the health sector, and among the transformation agendas quality and equity of services is the priority [14]. Hence, stressing the importance of recognizing where the country stands in terms of equity in the use of maternal health services.

Up until now the focus has been on improving access, coverage, health infrastructure and provision of quality service [20, 21]. Therefore, in order to address the underprivileged, it is crucial to investigate the extent of inequalities in utilization of reproductive and maternal health services. The objective of the study was to examine and measure inequalities in utilization of reproductive and maternal health services based on four socioeconomic stratifiers, administrative regions, urban/rural, education and economic status.

## Methods

### Country profile

Ethiopia is the second most populous country in Africa. According to the 2007 population and housing census projection, the total population is estimated to be 90 million by the year of 2015. The rural part of the country accounts for 84% of the Population [22]. The country is comprised of nine Regional States: Tigray, Amhara, Oromia, Southern Nation Nationalities and Peoples Region (SNNPR), Afar, Somali, and Harari; and two City Administrations council of Dire Dawa and Addis Ababa. Tigray, Amhara, Oromia and SNNPR are richer regions, while Afar, Somali and Harari are nomadic regions predominantly. Benishangul- Gumuz and Gambella are among the poorer regions in the country [23]. In terms of education, nowadays the net primary school enrolment (Grade 1–6) has reached 99% [14].

Currently, Ethiopia has one of world’s fastest growing economy. The economy has grown rapidly registering 10.9% GDP annual average growth rate over the past decade (2003/4 -2013/14). More than a decade ago, the country suffered from one of the highest poverty rates in the world, with majority of the population living below the international poverty line of $1.25 purchasing power parity (PPP) a day [24]. This poverty rate has dramatically reduced from 56% in 2000 to 29% in 2012 [24]. The county’s health care financing is highly dependent on donors (with almost 50% of total health expenditure), followed by households (34%) in forms of out-of-pocket expenditure at the point of care [25].

Ethiopia has a three-tier health care delivery system. The first level is a Woreda/District health system comprising a primary hospital, health centers and their satellite Health Posts. The first two for the most part are engaged in curative and preventive health services, while health posts are front-runners in provision of preventive health services with a special attention to maternal and child health. The second level in the tier is a General Hospital and the third is Specialized Hospital, both exclusively focusing on curative health services. In 2015, there were a total of 311 hospitals (private included), 3547 health centers and 16,440 health posts available in the country [14].

### Data sources

The study used data from 2014 Ethiopian demographic health survey for analysis. The survey was employed based on 2007 population and housing census of Ethiopia [22]. The survey included all 11 regions found in the country. All women of reproductive age (15–49 years) residing in the selected households were eligible for the survey. The sample was pulled in two stages. In the first stage, 305 sampling units were drawn with a probability proportional to the region’s size. Then, 30 households per cluster were selected through equal probability systematic selection technique. Finally, 8070 women of reproductive age group were interviewed out of the targeted 9150 eligible women.

### Study variable and definition

The variables under this study are described under Table 1

**Table 1:** Definition of reproductive and maternal health service indicators used in the study, 2014 Ethiopia

Maternal health services	Definition
Use of modern contraceptive methods	Percentage of currently married women age 15–49 utilizing modern contraceptive method
Antenatal care by skilled provider	Percentage of women age 15–49 who had a live birth in the 5 years preceding the survey that received antenatal care from physicians, nurses or midwives at least once
Skilled birth attendance	Percentage of births assisted by physicians, nurses, midwives or auxiliary midwives
Birth at health facility	Percentage of live births in the 5 years preceding the survey delivered in a health facility (private or public)
Postnatal care	Percentage of women age 15–49, with a live birth in the 2 years preceding the survey who received a postnatal checkup in the first 2 days after giving birth

#### Wealth index

Wealth index in the survey was computed using household asset data through principal component analysis. Wealth index is used as an indicator of level of wealth that is in line with income and expenditure measures. The wealth index in the survey was created in three steps. First, wealth scores were calculated using indicators common for both urban and rural areas. In the second step, using indicators specific to household’s in urban and rural areas separate factor scores were produced. Then in the third stage, separate area specific factor scores were combined to produce a nationally applicable wealth index by adjusting scores specific to the areas [26].

#### Levels of education

Educational status of women in the survey was categorized under four standard levels. Under the first category, women with no formal education labeled as no education. Women having (grade 1–8) formal education were categorized under primary level education. The third category includes, women having formal education of grade 9–12 considered as secondary level educated. Women receiving more than secondary education, those joining higher education and technical-professional education were labeled as more than secondary level in the survey [23].

#### Urban/rural

Urban areas include all capitals of administrative regions, zones and woredas. Areas with at least 1000 people primarily engaged in non-agricultural activities and/or localities declared as urban area by administrative officials. Rural areas are all areas which are not urban areas [27].

#### Skilled provider

Skilled health service providers includes doctor, nurse, midwife, and auxiliary nurse/midwife. Health extension workers are not considered as health professionals, but they are mainly involved in provision of promotive and preventive health services. Traditional birth attendants are birth attendants without any formal education background in service provision [28].

### Data analysis

Several methods are available to evaluate equity in healthcare utilization. Equity for each reproductive and maternal health services were estimated using four standard equity measures – equity gaps, equity ratios, concentration curves and concentration indices [28, 29]. The absolute percentage point difference in service coverage between the richest and poorest groups is used to show equity gaps. The equity ratio is calculated by dividing service coverage in the top wealth quintile by that in the bottom. The concentration curve plots the cumulative percentage of the population, ranked by wealth, beginning with the poorest, and ending with the richest (x-axis) against the cumulative percentage of the health service utilization (y-axis).

Concentration index (CI) is two times the area between the line of equality and the concentration curve. The index takes a value between −1 and +1; 0 index indicates presence of equality in utilization of the health variable. If socioeconomic inequalities exist, it can be seen in one of the two forms, the first and the most common is when there is uneven concentration of favorable health variable (access and utilization of health services) among the rich, in which case the concertation index takes on a positive value. The second is when the concentration index assumes negative value, which implies disproportionate concentration of health variable among the poor (commonly mortality and morbidity). When the concentration index takes on positive value the line on concentration curve will be below the equality line and vice versa [19–22]. The concentration index for t = 1, …,T groups is easily computed in a spreadsheet program using the following formula [29–32]:

$$ C=\left({p}_1{L}_2-{p}_2{L}_1\right)+\left({p}_2{L}_3-{p}_3{L}_2\right)+\dots +\left({p}_{T-1}{L}_T-{p}_T{L}_{T-1}\right) $$

where *p*_*T*_ is the cumulative percentage of the sample ranked by economic status in group t, and *L*_*T*_ is the corresponding concentration curve ordinate. Data was analyzed using STATA 13.0 and Microsoft Excel worksheet.

## Results

Equity of reproductive and maternal health services were assessed in terms of four socio-economic determinants; wealth quintile, maternal education, administrative region and place of residence. The use of reproductive and maternal health indicators is presented in Table 2.

**Table 2:** Utilization of major reproductive and maternal health services according to socio-determinants, Ethiopia 2014

Background characteristics	Use of modern contraceptive		Antenatal care by skilled provider		Birth at health facility		Skilled birth attendance		Postnatal care	
	Number	%	Number	%	Number	%	Number	%	Number	%
Residence										
Urban	882	55.6	521	80.3	681	58.5	681	58.4	260	47.8
Rural	4263	37.2	3157	34.8	4634	10.2	4634	9.1	1607	7.5
Regions										
Tigray	316	29.6	227	68.7	333	26.7	333	26.2	129	23.5
Affar	56	13.7	39	31.0	64	9.9	64	10.0	22	8.0
Amhara	1240	48.0	837	46.2	1120	12.0	1120	11.7	396	8.9
Oromiya	2046	39.1	1471	32.7	2215	13.3	2215	13.1	784	10.9
Somali	104	1.6	98	19.1	188	15.9	188	15.3	62	3.7
Benishangul-Gumuz	54	38.8	39	38.8	59	21.0	59	16.3	22	19.8
SNNP	1077	39.2	819	39.0	1151	14.9	1151	11.7	379	11.1
Gambela	30	50.4	19	54.2	25	31.9	25	29.1	8	15.8
Harari	14	42.9	9	69.7	12	45.3	12	45.5	5	39.3
Addis Ababa	184	57.4	103	94.2	125	86.5	125	86.1	53	70.1
Dire Dawa	22	34.6	15	78.4	22	59.2	22	59.2	8	49.3
Education										
No education	3121	34.6	2302	32.0	3452	8.8	3581	7.5	1137	8.1
Primary	1586	46.0	1137	50.5	1582	22.5	1542	21.0	597	15.0
Secondary	282	58.9	168	81.9	194	69.0	170	69.4	85	42.0
More than secondary	156	64.7	71	96.3	87	91.7	77	90.7	48	59.9
Economic status										
Lowest	1001	27.1	836	23.7	1276	5.1	1276	4.5	456	3.4
Second	1046	36.3	778	29.3	1146	6.4	1146	5.5	365	4.5
Middle	1009	37.3	726	40.6	1103	11.0	1103	9.1	383	12.7
Fourth	970	46.3	680	42.2	961	15.1	961	14.5	340	9.9
Highest	1119	53.6	657	77.3	829	56.5	829	55.6	325	40.6
Overall	5245	40.4	3678	41.2	5315	16.4	5315	15.5	1868	13.2

Source of data: Ethiopia DHS 2014

### Use of modern contraceptive methods

There was no significant difference in use of contraceptive methods among women in the nine administrative regions of the country. However, Somali (1.6%) and Afar (13.7) were the two regional states utilizing the services at lower rate. Modern contraceptive use among currently married women living in urban areas is 1.5 times higher than in rural area. The prevalence among mothers with no education is about two times less than those with more than secondary educated.

### Antenatal care

ANC is considered as one of the major indicators for pregnant women to give birth at health facility. Somali regional state has the lowest ANC service utilization (19.9%), while the capital city (Addis Ababa) had the highest (94.2%) utilization rate. ANC service use is 2.3 times, higher in the urban area than rural area. Educational status of mothers is also major socio-economic factor affecting utilization of ANC service. It is 3 times higher among women with more than secondary level educated than among mothers with no education. ANC service by skilled provider is 53.6% higher in the richest quantile than in the poorest quantile. On the contrary, ANC service provided by health extension workers (HEWs) is 13.1% higher in the poorest quantile than in richest quantile.

### Place of delivery

Among the nine regions and two administrative cities, the Afar region had the lowest utilization (10%) and Addis Ababa had the highest utilization (86.5%). Women from urban area had 5.7 times more birth at health facility than women from rural area. Facility based delivery was 2.8 times higher in primary level educated women than those with no education. Mothers with more than secondary educational status had 4.3 times higher facility based delivery than those with primary level education. Women in the secondary level education category had 9.3 times higher facility based delivery than those with no education. Majority of the mothers (84%) gave birth to their child at home, mainly assisted by relatives and traditional birth attendants (TBA). Some were assisted by skilled birth attendants of which majority were economically better-offs.

### Postnatal care

Like that of facility-based delivery, women receiving postnatal checkup is very low. Once again, the Somali region has the lowest utilization (3.7%), while the capital city Addis Ababa had the highest utilization (70.1%). Women residing in the urban area had 6.4 times higher PNC uptake than those living in rural areas. Like other maternal health services discussed above PNC is also pretty much affected by maternal education. Women with more than secondary level of education had 7.4 times higher PNC uptake than mothers with no education.

**Fig. 1:**
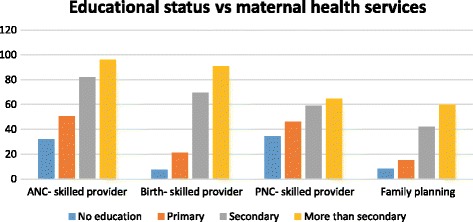
Utilization of maternal health services by maternal education, Ethiopia 2014

**Fig. 2:**
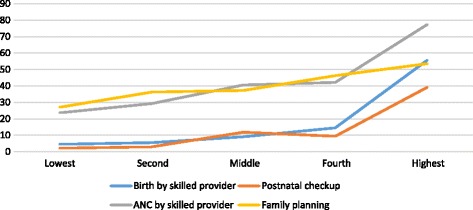
Maternal health services by wealth quantile, Ethiopia 2014

**Fig. 3:**
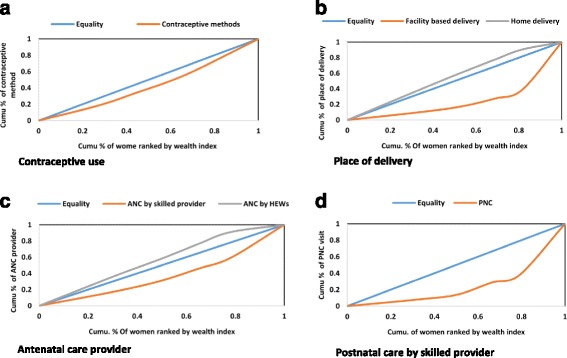
Concentration curve for indicators of reproductive and maternal health service, 2014 Ethiopia

In general, in addition to urban rural disparities, two key socio-economic factors were found to play a significant role for mothers to attend maternal health services (See Figs. 1 and 2). Concentration curve for the investigated reproductive and maternal health services revealed pro-rich bias (see Fig. 3). In addition, Table 3 presents results of rate-ratios and concentration indices with standard error and confidence intervals based on economic status of the women, justifying inequality in utilization of maternal health services.

**Table 3:** Rate-ratio (rich/poor), Concentration indices standard error and confidence interval for maternal health services, Ethiopia 2014

Indicators	Rate-ratio (rich/poor)	CI	Standard error	Confidence interval (95%)	
				Lower	Upper
Use of modern contraceptive^*^	1.98	0.143	0.032	0.080	0.206
ANC by skilled provider	3.26	0.251	0.29	-0.317	0.819
Antenatal care by HEW	0.345	-0.135	0.095	-0.321	0.051
No Antenatal care^*^	0.281	-0.195	0.082	-0.356	-0.034
Delivery at home	0.45	-0.107	0.065	-0.234	0.020
Facility based delivery^*^	11.1	0.502	0.039	0.426	0.578
Delivery by skilled provider^*^	12.36	0.525	0.042	0.443	0.607
Delivery by TBA^*^	0.403	-0.161	0.051	-0.261	-0.061
Delivery: relative/other	0.45	-0.072	0.083	-0.235	0.091
Postnatal checkup	11.9	0.493	0.59	-0.663	1.649

*Significant at *P* < 0.05

## Discussion

This study attempted to assess inequities in utilization of reproductive and maternal health services in Ethiopia. Even though there were encouraging progresses in the past five years, maternal health service indicators are still alarmingly low and exhibit significant disparities between and within regions of Ethiopia.

Two developing regions in Ethiopia, Somali and Afar have the lowest level of modern contraceptive service utilization. These are also among the regions with the lowest human development index [23]. The rate of modern contraceptive methods in women at the top of wealth quantile is about two fold that of women at the bottom of wealth quantile. The computed concentration index also justifies the same finding (CI; 0.143). However, a study in Thailand [22] indicated that the rate of modern contraceptive methods among women at the bottom of wealth quantile is 1.01 times the women at the top of wealth quantile. The CI was -0.009, which meant the services were more utilized by the poor. Another study in Cambodia indicated the rate of met needs for family planning was 1.33 times higher among the economically better-off than the poor [33].

ANC service is by far utilized by pregnant women living in urban areas than those living in rural areas, educated women than women with no education, and by richer women than poorer women. Rate of ANC service is 3.26 times, higher in women at the top quantile than in the bottom wealth quantile. The concentration index was 0.251, which implies that the rich mainly utilize ANC service. This finding is higher than the one reported from Namibia [30], rate-ratio of 1.06 and CI of 0.0130. Even though not considered as skilled providers, preventive and promotive health services are provided by health extension workers (HEWs). ANC is one of the services provided by HEWs. Less educated pregnant women than educated, those living in rural area than urban area and those at the bottom quantile than top quantile, mainly utilize ANC service by HEWs. It is almost the opposite of ANC provided by skilled providers (Doctors/Nurses/Midwifes). Here, the rate of ANC is 2.89 times, higher among the poor than that of the economically better-off. The CI is -0.135, indicating that, the service is the mainly utilized by the poor. This could be due to the fact that HEWs are the first contact to health care system in Ethiopia especially in rural areas, where 85% the population resides [23]. HEWs are stationed closer to the population, besides working at their post they also go house to house and provide basic preventive and health promotion services [34]. As a result, they can reach part of the population who are unable to visit health centers or hospitals. Mothers at top wealth quintile have 12.3 times higher skilled provider assistance during birth than mothers at the bottom of wealth quantile. This finding is far worse than the study from Namibia where the rate is 1.63 times, higher among the rich than the poor, with CI of 0.0943 [30]. The computed CI: 0.525 also implied that the service is highly favored by the economically better off. Nowadays, many health centers are constructed in most parts of rural Ethiopia. However, urban/rural disparities continue to persist partly owing to the long distance between health facilities and residential places of the women [35]. The computed rate-ratios revealed that the uptake of birth by skilled provider is 6.4 times, higher among women living in urban areas than those living in rural areas. The government proclaims that majority of maternal health services, especially labor and delivery is free of payment, but many of the health facilities receive payments for certain aspects of the services [15].

Majority of maternal mortality occurs during postpartum period [36, 37]. While postnatal care has been proven to reduce maternal mortality during this period [38], only 13% of women received postnatal care within the first two days of delivery.

The computed CI: 0.493 revealed that the wealthy segment of the population mainly used the service. The rate of PNC in women among top wealth quantile was about 12 times that of women at the bottom of wealth quantile. When compared to other studies from Namibia [30] with CI of 0.0835, the finding of this study is high. This paper supports the commonly known, robust and persistent associations of social determinants of health, especially economic status and level of education with utilization of important maternal health services as stated by Ahmed et al. [4].

Educating women enhances their health- seeking behaviors, and there by improving the need to attend maternal health care services. Therefore, strengthening inter-sectoral collaboration among development sectors is crucial in order to improve maternal health and promote equity. This also means that educating women and reducing poverty, so that we can see dramatic improvements in maternal health [39]. While improving quality of services and increasing coverage are work in progress in Ethiopia, in places where health services are accessible, they often fall short of being patient-friendly.

Recently, Yesuf et al. stated that developing regions, especially Somali and Afar have the lowest birth at health facility and antenatal care utilization, which is in line with this study [10, 11]. Possible explanation for these findings could be the poorly developed health system infrastructure and low capacity to set priorities and allocate available resources in these emerging regions of the country [14, 24]. It is also likely that the disparity found in these regions relates to low literacy rate of the population that affects the use of available maternal health services.

Limitations the study may include, interpretation of the findings we have reported in this article should take note of the fact that the analysis in the current study is unable to reflect the current level of utilization and disparities in utilization owing to the time of data generation. Moreover, data collected on the survey were based on use of maternal health services in the past 2–5 years, which introduces the possibility of recall bias in this study.

## Conclusion

Significant inequity between and within regions of Ethiopia in the use of maternal health services was demonstrated. Strategically targeting social determinants of health with special emphasis to women education and economic empowerment will significantly contribute for altering the current situation favourably. Geographical disparities in terms of regional and urban/rural differences in the use of maternal health services should be addressed by actively mobilizing the communities to raise awareness and use and making sure that the arrangements for service fit to the disadvantaged segments of the population. Finally, we recommend conducting decomposition of concentration index analysis to explain socioeconomic-related health inequalities.

## Abbreviations

ANC: Antenatal care
CI: Concentration index
DHS: Demographic and health survey
HEWs: Health extension workers
PNC: Postnatal care
TBA: Traditional birth attendance
WHO: World Health Organization

## References

[1] World Health Organization (2010). The world health report 2010: health systems financing: the path to universal coverage.

[2] Houweling T, Ronsmans C, Campbell O, Kunst A (2007). Huge poor-rich inequalities in maternity care: an international comparative study of maternity and child care in developing countries. Bull World Health Organ.

[3] Culyer AJ, Wagstaff A (1993). Equity and equality in health and health care. J Health Econ.

[4] Ahmed S, Creanga AA, Gillespie DG, Tsui AO (2010). Economic status, education, and empowerment: implications for maternal health service utilization in developing countries. PLoS One.

[5] Alam N, Hajizadeh M, Dumont A, Fournier P (2015). Inequalities in maternal health care utilization in Sub-saharan african countries: a multiyear and multi-country analysis. PLoS One.

[6] Alkenbrack S, Chaitkin M, ZengW CT, Sharma S (2015). Did equity of reproductive and maternal health service coverage increase during the MDG Era? an analysis of trends and determinants across 74 Low- and middle-income countries. PLoS One.

[7] Say L, Raine R (2007). A systematic review of inequalities in the use of maternal health care in developing countries: examining the scale of the problem and the importance of context. Bull World Health Organ.

[8] Campbell OMR, Graham WJ (2006). Strategies for reducing maternal mortality: getting on with what works. Lancet.

[9] Gabrysch S, Campbel O (2009). Still too far to walk: literature review of the determinants of delivery service use. BMC Pregnancy Childbirth.

[10] Yesuf EA, Kerie MW, Calderon-Margalit R (2014). Birth in a health facility –inequalities among the ethiopian women: results from repeated national surveys. PLoS One.

[11] Yesuf EA, Calderon-Margalit R (2013). Disparities in the use of antenatal care service in Ethiopia over a period of 15 years. BMC Pregnancy Child Birth.

[12] Ambel, Alemayehu; Andrews, Colin; Bakilana, Anne; Foster, Elizabeth; Khan, Qaiser; Wang, Huihui. Maternal and Child Health Inequalities in Ethiopia. Policy Research Working Paper;No. 7508. World Bank, Washington, DC; 2015. 10.1186/s12939-017-0648-1 PMC5568328 28830454

[13] Wabiri N, Chersich M, Zuma K, Blaauw D, Goudge J (2013). Equity in maternal health in south africa: analysis of health service access and health status in a national household survey. PLoS One.

[14] Ministry Of Health (Federal Democratic Republic of Ethiopia) (2015). Health sector transformation plan.

[15] Pearson L, Gandhi M, Admasu K, Keyes EB (2011). User fees and maternity services in Ethiopia. Int. J. Gynaecol. Obstet..

[16] Ganle JK, Parker M, Fitzpatrick R, Otupiri E (2014). Inequities in accessibility to and utilisation of maternal health services in Ghana after user-fee exemption: a descriptive study. Int J Equity Health.

[17] Simkhada B, van Teijlingen E, Porter M, Simkhada P (2008). Factors affecting the utilization of antenatal care in developing countries: systematic review of the literature. J. Adv. Nurs..

[18] Woldemichael G, Tenorang EY (2010). Women’s autonomy and maternal health seeking behavior in Ethiopia. Matern. Child Health J..

[19] Ministry of Health (Federal Democratic Republic of Ethiopia) (2007). Health extension programme in Ethiopia.

[20] Ministry of Health (Federal Democratic Republic of Ethiopia) (2010). Health sector development program IV.

[21] Ministry of Health (Federal Democratic Republic of Ethiopia) (2009). Health sector strategic plan III.

[22] Central Statistical Agency [Ethiopia] (2008). The 2007 population and housing census of ethiopia.

[23] United Nations Development Programme (2015). National human development report 2014 ethiopia.

[24] World Bank Group. Ethiopia Poverty Assessment 2014. World Bank, Washington, DC; ​2015

[25] Ethiopia Federal Ministry of Health. Ethiopia’s Fifth National Health Accounts 2010/2011. Addis Ababa: Ministry of health-Ethiopia; ​2014.

[26] Central Statistical Agency [Ethiopia]: Ethiopia Demographic and Health Survey 2014. Addis Ababa, Ethiopia: Central Statistical Agency; August 2014.

[27] Schmidt E, Kedir M. Urbanization and Spatial Connectivity in Ethiopia: Urban Growth Analysis Using GIS. ESSP II Work Pap. 2009;3. Available from: http://essp.ifpri.info/files/2011/02/ESSP2_DP03_Urbanization-and-Spatial-Connectivity-in-Ethiopia.pdf.

[28] Barros AJ, Victora CG (2013). Measuring coverage in MNCH: determining and interpreting inequalities in coverage of maternal, newborn, and child health interventions. PLoS Med..

[29] O’Donnell O, van Doorslaer E, Wagstaff A, Lindelow M. Analyzing Health Equity Using Household Survey Data: A Guide to Techniques and Their Implementation. World Bank.Washington, DC; 2008.

[30] Zere E, Tumusiime P, Walker O, Kirigia J, Mwikisa C, Mbeeli T (2010). Inequities in utilization of maternal health interventions in Namibia: implications for progress towards MDG 5 targets. Int J Equity Health.

[31] Zere (2013). Equity in reproductive and maternal health services in Bangladesh. Int J Equity Health.

[32] Zere E, Moeti M, Kirigia J, Mwase T, Kataika E (2007). Equity in health and healthcare in Malawi: analysis of trends. BMC Publ Health.

[33] Dingle (2013). A decade of improvements in equity of access to reproductive and maternal health services in Cambodia, 2000–2010. Int J Equity Health.

[34] Central Statistical Agency [Ethiopia] and ICF International: Ethiopia Demographic and Health Survey 2011. Addis Ababa, Ethiopia and Calverton, Maryland, USA: Central Statistical Agency and ICF International; 2012.

[35] Bailey PE, Keyes EB, Parker C, Abdullah M, Kebede H (2011). Using a GIS to model interventions to strengthen the emergency referral system for maternal and newborn health in Ethiopia. Int J Gynecol Obstet.

[36] Khan KS, Wojdyla D, Say L, Gülmezoglu AM, Van Look PFA (2006). WHO analysis of causes of maternal death : a systematic review. Lancet.

[37] Ronsmans C, Graham WJ; Lancet Maternal Survival Series Steering Group. Maternal mortality: who, when, where and why. Lancet 2006;368:1189–200. doi:10.1016/S0140-6736(06)69380-X PMID:17011946 10.1016/S0140-6736(06)69380-X 17011946

[38] Shaw E, Levitt C, Wong S. Systematic Review of the Literature on Postpartum Care : Effectiveness of Postpartum Support to Improve Maternal Parenting, Mental Health, Quality of Life, and Physical Health. Birth 2006;210–20. 10.1111/j.1523-536X.2006.00106.x 16948721

[39] Marmot M, Friel S, Bell R, Houweling TA, Taylor S, Commission on social determinants of health (2008). Closing the gap in a generation: health equity through action on the social determinants of health. Lancet.

